# New‐Onset Urethral Stricture After HoLEP: Incidence, Predictors and Treatment Outcomes From an 8‐Year Single‐Centre Analysis

**DOI:** 10.1002/bco2.70251

**Published:** 2026-07-15

**Authors:** Juanita Velasquez‐Ospina, Gurpremjit Singh, Ahmad Abdelaziz, Adam Williams, Haşim Bakbak, Jonathan Katz, Robert Marcovich, Hemendra N. Shah

**Affiliations:** ^1^ Desai Sethi Urology Institute, Miller School of Medicine University of Miami Miami Florida USA

**Keywords:** BPH, complications, endoscopic prostate surgery, HoLEP, urethral stricture

## Abstract

**Objectives:**

The objective of this study is to evaluate the incidence, timing, anatomical characteristics, predictors and management outcomes of urethral stricture formation following HoLEP at an academic institution in the United States.

**Materials and Methods:**

A retrospective review was conducted of 857 patients who underwent HoLEP at a single academic institution between July 2017 and August 2025. Patients with prior or incidental urethral stricture diagnosis at the time of HoLEP were excluded. Enucleation was performed using a 26‐Fr resectoscope, and morcellation was completed through a 26‐Fr morcelloscope sheath. If initial cystoscopy suggested small‐sized meatus or non‐distensible penile urethra, Otis urethrotomy or urethral calibration was performed. Demographic, clinical and perioperative data were collected and analysed using multivariable logistic regression to identify predictors of postoperative urethral stricture formation.

**Results:**

Among 857 patients, 16 had preoperative or intraoperative strictures and were excluded. Seventeen patients (2.0%) developed urethral stricture at a mean of 6.2 months postoperatively. Patients with stricture had lower BMI (24.1 vs. 27.4 kg/m^2^, *p* = 0.0042) and smaller prostates (65 vs. 105 ml, *p* = 70.0131). Median age was higher in the stricture group (73 vs. 69 years, *p* = 0.0702). On multivariable analysis, higher BMI was independently associated with lower odds of postoperative urethral stricture. (OR 0.84, 95% CI 0.73–0.97; *p* = 0.016). Stricture locations included bulbar (52.9%), bulbo‐membranous (11.8%), sub‐meatal (11.8%), penile (5.9%), membranous (5.9%), prostatic (5.9%) and combined bulbar and sub‐meatal (5.9%). Most strictures (76.5%) were <1 cm. Initial management included laser incision (seven), dilation (nine) and drug‐coated balloon dilation (one). At mean post‐treatment follow‐up of 17.53 months, four patients (23.5%) experienced recurrence of stricture that required additional management.

**Conclusion:**

The overall risk of urethral stricture following HoLEP was low (2%). Higher BMI was associated with a lower risk of urethral stricture formation. Following initial intervention, 23.5% experienced relapse needing additional management.

## 1 INTRODUCTION

Holmium laser enucleation of the prostate (HoLEP) is an established, size‐independent surgical treatment for benign prostatic obstruction (BPO).^1^, ^2^ Its adoption in the United States has steadily increased, rising from 4.8% to 7.6% between 2015 and 2020, reflecting favourable functional outcomes, long‐term durability and an acceptable safety profile across a wide range of prostate sizes, including patients with coagulopathies.^3^, ^4^

Despite its favourable safety profile, HoLEP is not without complications. Urethral stricture, although uncommon, represents a clinically meaningful adverse outcome, with reported rates ranging from 1% to 5%.^5^, ^6^, ^7^ Even when infrequent, post‐HoLEP urethral strictures may result in recurrent obstruction, incomplete symptom resolution or worsening lower urinary tract symptoms, often necessitating additional intervention.^6^, ^8^

The pathophysiology of urethral stricture formation following HoLEP is not fully understood. Proposed mechanisms include mechanical trauma and ischemic injury related to the passage of large‐calibre endoscopic instruments, leading to urothelial injury, fibrosis and subsequent luminal narrowing.^9^, ^10^ Prior studies have reported associations between stricture formation and smaller preoperative prostate volumes, lower resected tissue weight and longer operative duration.^5^, ^6^, ^11^ However, the existing literature remains limited by heterogeneity in outcome definitions, including combined reporting of urethral strictures and bladder neck contractures and inclusion of intraoperatively identified strictures. Additionally, there is limited data characterizing the timing, anatomical location, clinical presentation and management outcomes of urethral strictures following HoLEP, which restricts the ability to accurately counsel patients and identify those at increased risk.

The objective of this study was to evaluate the incidence, timing, anatomical characteristics, predictors and management outcomes of urethral stricture formation following HoLEP at an academic institution in the United States. By providing detailed characterization of post‐HoLEP urethral strictures and their treatment outcomes, this study aims to address important gaps in the literature, improve risk stratification and support more informed patient counselling and postoperative management.

## 2 METHODS

Following IRB approval, we retrospectively reviewed patients who underwent HoLEP at an academic centre between July 2017 and August 2025. Medical charts were reviewed manually to identify postoperative urethral strictures. Patients who developed bladder neck contractures were evaluated independently and were not included in the urethral stricture outcome analysis. Patients with a history of strictures or those with intraoperative findings of stricture including those with non‐compliant, rigid or stiff urethra secondary to fibrosis were excluded. Patients with a prior history of pelvic/prostate radiation therapy were included, reflecting the complex referral population encountered at our tertiary referral academic centre.

All procedures were performed or supervised by a single surgeon (HS) with experience of over 1600 HoLEP before the start of the study period. The en‐bloc HoLEP technique was employed with a 26‐Fr endoscope followed by morcellation through a 26‐Fr morcelloscope sheath. In the last 2 years, we modified the technique attempting to preserve anterior fibromuscular stroma.^12^ Urethral calibration or Otis urethrotomy was performed at the surgeon's discretion if a small size meatus or non‐distensible penile urethra was encountered during initial cystoscopy. Postoperatively, a 22‐Fr Foley catheter was placed without traction, and continuous bladder irrigation was maintained overnight, with discharge planned on postoperative Day 1 following a successful voiding trial.

Demographic and perioperative variables collected included age, comorbidities, prostate volume, history of clean intermittent catheterization (CIC), prior BPH surgery, history of radiation therapy, preoperative catheterization status, preoperative urinary retention, operative duration, duration of postoperative catheterization and prostate cancer detection on histopathological exam of prostate specimen. All patients were asked to come for postoperative follow‐up at 6 weeks, 3 months, 6 months and 12 months postoperatively and annually thereafter. At each visit, patients had IPSS, uroflowmetry and postvoid residual urine estimation. Those with symptoms or uroflow suggesting urethral stricture or bladder neck stenosis underwent further evaluation with retrograde urethrogram (RUG) and/or cystoscopy. Routine cystoscopy was not performed on all patients. Stricture length was classified as <1 or ≥1 cm based on cystoscopic and/or radiographic assessment. Stricture characteristics, management and recurrence were recorded. Patients who were managed for postoperative urethral stricture were contacted by telephone to update their symptom status and need for additional intervention for possible stricture recurrence.

Continuous variables were summarized using medians and interquartile ranges and compared using Wilcoxon rank‐sum tests. Categorical variables were reported as frequencies and percentages and compared using a Chi‐Square test or Fisher's exact test, as appropriate. Univariable and multivariable logistic regression analyses were performed to identify predictors of urethral stricture formation. Variables included in the multivariable model were selected based on statistical significance and clinical relevance in univariable analysis. Statistical significance was defined as *p* < 0.05. All analyses were conducted using SAS Version 9.4.

## 3 RESULTS

### 3.1 Patient characteristics

A total of 857 patients underwent HoLEP at our centre in the study period. Sixteen patients with a preoperative diagnosis or intraoperative incidental urethral strictures were excluded. Among the 841 remaining, 17 (2%) developed postoperative urethral strictures at a mean of 6.2 months (range: 4.4–13.6).

### 3.2 Patient and perioperative characteristics

Baseline demographic and clinical characteristics were largely comparable between patients with and without stricture (Table 1). Patients who developed strictures had significantly lower BMIs and smaller preoperative prostate volumes. One patient in the stricture group had a prior history of radiation therapy for prostate cancer. Five patients in our study needed use of electrocoagulation for haemostasis after HoLEP, and only one patient needed to be taken back to the OR for clot evacuation. None of these six patients developed postoperative urethral strictures.

**TABLE 1:** Patient characteristics and multivariable logistic regression for predicting urethral stricture after HoLEP.

	Overall cohort (*n* = 841)	No stricture (*n* = 824)	Stricture (*n* = 17)	*p* value
Age (median, IQR)	69 (63, 76)	69 (63, 76)	73 (67, 80)	0.0702
BMI	27.3 (24.7, 30.4)	27.4 (24.8, 30.6)	24.1 (23.1, 26.0)	**0.0042**
PSA	4.6 (2.3, 8.1)	4.7 (2.4, 8.1)	2.6 (0.9, 8.4)	0.1133
Prostate volume	103 (75, 156)	105 (75, 156)	65 (40, 110)	**0.0131**
PSA density	0.04 (0.03, 0.07)	0.04 (0.03, 0.07)	0.04 (0.02, 0.07)	0.8559
Comorbidities (*n*, %)				
Diabetes mellitus	210 (25.0%)	204 (24.8%)	6 (35.3%)	0.3205
Hypertension	451 (53.6%)	442 (53.6%)	9 (52.9%)	0.9543
Dyslipidemia	386 (45.9%)	376 (45.6%)	10 (58.8%)	0.2799
Catheterization	267 (31.8%)	263 (31.9%)	4 (23.5%)	0.4621
Hx CIC	74 (8.8%)	72 (8.7%)	2 (11.8%)	0.6628
Hx of BPH surgery	116 (13.8%)	114 (13.8%)	2 (11.8%)	0.8916
More than 2 BPH surgeries	26 (3.1%)	25 (3.0%)	1 (5.9%)	0.5018
History of radiation therapy	10 (1.18%)	9 (1.1%)	1 (5.9%)	0.184
Retention status				
None	187 (22.2%)	182 (22.1%)	5 (29.4%)	0.337
Chronic	490 (58.3%)	479 (58.1%)	11 (64.7%)	
Acute	164 (19.5%)	163 (19.8%)	1 (5.9%)	
Duration of surgery (minutes)	147 (107, 195)	147 (107, 194)	132 (111, 230)	0.9681
Duration of postoperative catheterization (median, IQR)	1 (1, 2)	1 (1, 2)	1 (1, 1)	0.051
Need for prolonged postoperative catheterization (>1 week), *n* (%)	113 (13.4%)	112 (13.4%)	1 (5.9%)	0.714
Histopathology of PCa	103 (12.1%)	100 (12.5%)	3 (17.7%)	0.4927
Time from surgery to stricture (months)	N/A	N/A	6.2 (4.4, 13.6)	–

Multivariable analysis		
	OR (95% CI)	*p* value
Age (per year)	1.03 (0.98, 1.09)	0.28
Prostate Size (per cc)	0.99 (0.98, 1.00)	0.059
BMI (per unit)	0.84 (0.73, 0.97)	**0.016**

*Note*: Bold text highlights statistically significant findings (*p* < 0.05).

Abbreviations: BPH, benign prostatic hyperplasia; CIC, clean intermittent catheterization; Hx, history; PCa, prostate cancer; PSA, prostate specific antigen.

### 3.3 Stricture characteristics and management

Nine patients developed bulbar strictures (52.9%), two bulbo‐membranous (11.8%), two sub‐meatal (11.8%), one penile (5.9%), one membranous (5.9%), one prostatic (5.9%) and one with both bulbar and sub‐meatal (5.9%). The patient with prostate urethral stenosis had a history of transurethral water vapour therapy prior to HoLEP. Because he had an open bladder neck, he was included in this study. Fifteen strictures were <1 cm (including the patient with two separate strictures), and three were 1–2 cm. No patients had strictures >2 cm in length. In addition to strictures, three patients also developed BNC following HoLEP; all managed at the same time as the urethral strictures, one managed with bladder neck incision and two with bladder neck resection. Mean follow‐up after stricture diagnosis was 17.5 ± 19.6 months and median follow‐up was 9 months (IQR 4–26); one patient was lost to follow‐up after diagnosis (5.8%).

**FIGURE 1:**
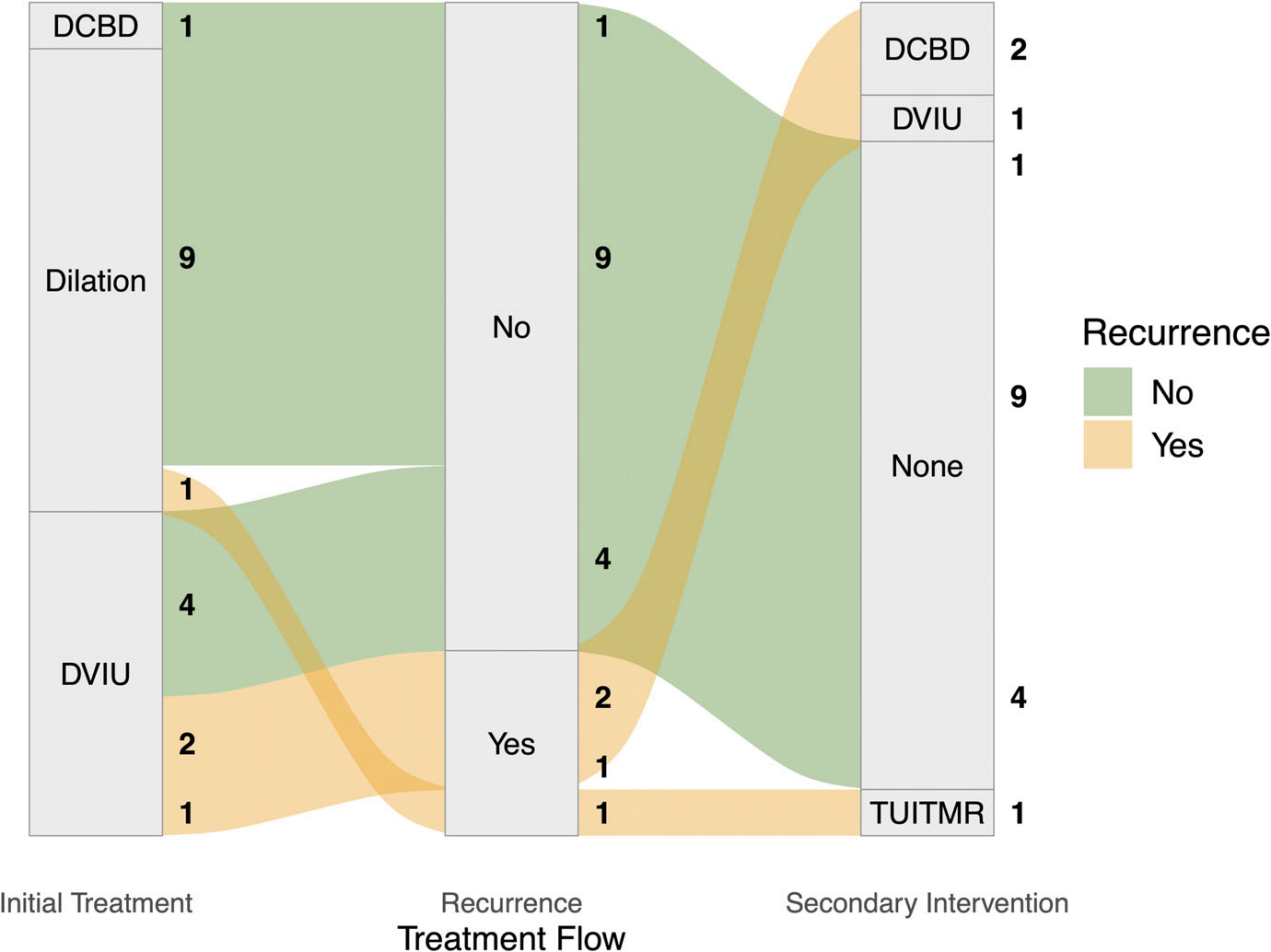
Flow of post‐HoLEP urethral stricture treatments from initial intervention to recurrence status and subsequent secondary interventions. DCBD, drug‐coated balloon dilation; DVIU, direct visual internal urethrotomy; TUITMR, transurethral incision with mucosal realignment *Total number of strictures exceeds the number of patients because we accounted for one patient who had two separate strictures.

Initial stricture management consisted of urethral dilation in nine patients (53%), direct visual internal urethrotomy (DVIU) in seven patients (41.2%) and primary drug‐coated balloon dilation in one patient (5.9%); the patient with two strictures was managed with dilation for both. Stricture recurrence occurred in four patients (23.5%) (Figure 1). Among these, one patient initially treated with dilation subsequently underwent transurethral ventral inlay buccal mucosa graft urethroplasty as described earlier^13^; two patients initially treated with DVIU required drug‐coated balloon dilation; and one patient required two DVIUs followed by drug‐coated balloon dilation. The patient who underwent primary drug‐coated balloon dilation did not experience recurrence during follow‐up.

### 3.4 Multivariable analysis

On multivariable logistic regression analysis, higher BMI was independently associated with lower odds of postoperative urethral stricture.

## 4 DISCUSSION

In this large cohort from an academic institution in the United States, the incidence of urethral stricture following HoLEP was low at 2%, consistent with prior reports and reinforcing the overall safety of the procedure with respect to long‐term complications.^14^ Elsaqa et al.^5^ and Thai et al.^15^ reported low urethral stricture rates following HoLEP of 1.94% and 2.98%, respectively (Table 2). More recent work by Taychert et al.^11^ surprisingly demonstrated a very low incidence of 0.44% in a large cohort of 1374 patients. In contrast, Glienke et al.^6^ reported a notably higher incidence of 5% despite a similarly large cohort of 1512 patients, which may reflect their extended follow‐up duration of 5 years. Across these studies and ours, operative techniques, instrumentation and perioperative practices were largely comparable, with similar sheath sizes and selective use of urethral dilation or urethrotomy based on surgeon preference (Table 2), suggesting that differences in reported stricture rates are less likely attributable to technical factors alone. It is worth mentioning that the overall incidence of stricture after HoLEP was not different from that noted after TURP.^16^

**TABLE 2:** Summary of published studies reporting urethral stricture after HoLEP.

Authors; year of publication; country; study design; total *n*		Elsaqa et al.; 2022; United States; retrospective review total *n* = 566	Glienke et al.; 2025; Germany; RR total *n* = 1512	Taychert et al.; 2025; United States; RR total *n* = 1374	Present study total *n* = 857
Stricture data		*n* = 19 (3.4%) 11 de novo, 8 intraop *Included preop and intraop US and BNCs in the risk factor analysis	*n* = 76 (5%) *Did not include preop US but does not specify if they included intraop US	*n* = 6 (0.44%) *Included BNCs in the risk factor analysis	*n* = 17 *Did not include intraop or preop US or BNCs
Preop urethral calibration/urethrotomy/dilation		Routine calibration up to 28Fr, Otis urethrotomy if according to surgeon preference	Not specified	Dilation up to 30Fr according to surgeon preference	Urethral calibration or Otis urethrotomy performed at surgeon's discretion
Preop indwelling catheter	No US	143 (26.5%)	457 (32.2%)	0 (0%)	263 (31.9%)
	US	5 (18.5%)	11 (14.4%)	16 (2.5%)^a^	4 (23.5%)
Preop prostate size (grammes)	No US	90 (60–122)	93.8 ± 47.2	103.9 ± 61.2	105 (75, 156)
	US	73 (61–105)	82.8 ± 39	72.6 ± 33.9	65 (40, 110)
OP time or ET/MT (min)	No US	OP time: 51 (34–71)	OP time: 79.9 ± 33.6	ET: 52.9 ± 26.9; MT: 11.8 ± 11.5	147 (107, 194)
	US	OP time: 47.5 (27–65)	OP time: 79.6 ± 33.5	ET: 39.4 ± 15.3; MT: 7.80 ± 8.50	132 (111, 230)
Resectoscope size		26 or 28 Fr	26 Fr	26 or 28 Fr	26 Fr
Stricture location		Bulbar 45%; penile 30%, fossa navicularis 15%, bulbo‐membranous 10%	Bulbar urethra (90.7%), penile urethra (27.6%), posterior urethra (5.2%) *15.7% multifocal	Meatal (16.7%), Fossa navicularis (50%), bulbar (33.3%)	Bulbar (52.9%); bulbo‐membranous (11.8%); sub‐meatal (11.8%); penile (5.9%); membranous (5.9%); prostatic (5.9%); bulbar and sub‐meatal (5.9%)
Postop time to diagnosis; total f/u after HoLEP*		50% at 6 weeks, 30% from 6 weeks to 6 month, 20% > 6 months F/u: median 20 months post‐HoLEP	11.9 months F/u: 5.03 years (range: 2.14–9 year)	148 days (range 75–205) US group: 26.7 ± 13.5 months Non‐US group: 6.5 ± 12.2 months	6.2 months F/u: mean 17.5 ± 19.6 months after US diagnosis
Initial intervention		70% dilation, 30% DVIU	Not specified	12% DVIU, 35.7% dilation, 7.1% urethroplasty, 7.1% meatotomy, 2.4% other^b^	Dilation 53%, DVIU 41.2%, drug‐coated balloon dilation 5.9%^c^
Significant differences/factors associated with strictures		Intraop stricture (*p* = 0.0055, OR = 15.5, 95% CI = 2.2–37.7), intraop need for meatotomy (*p* = 0.0019, OR = 7.69, 95% CI = 2.12–27.8), longer operative time (*p* = 0.0250, OR = 1.043, 95% CI = 1.005–1.083) were all factors associated with postoperative US and BNC	Preop indwelling catheter (OR 0.36, *p* = 0.002) was a protective factor against US	Lower prostate volume (OR 0.987, 95% CI: 0.974–0.997; *p* < 0.01) was associated with US	BMI was independently associated with lower odds of postoperative US (OR 0.84 95% CI 0.73–0.97; *p* = 0.016)

Abbreviations: BNC, bladder neck contracture; CI, confidence interval; DVIU, direct visual internal urethrotomy; ET, enucleation time; f/u, follow‐up; Intraop, intraoperative; MI, myocardial infarction; MT, morcellation time; OP, operative time; OR, odds ratio; Preop, preoperative; RR, retrospective review; US, urethral stricture.

^a^ Taychert et al. reported this variable as ‘catheter dependent’ versus ‘catheter independent’.

^b^ These values represent all surgical interventions performed during follow‐up and are not limited to initial management. Percentages do not sum to 100% because procedures for bladder neck contracture were also included and account for the remaining 35.7%.

^c^ The patient with two strictures was managed with dilation for both. This explains the percentages adding up to over 100%.

Urethral strictures were diagnosed at a mean of 6.2 months following HoLEP in our cohort. This is similar to Taychert et al.'s^11^ findings, who reported a mean time to diagnosis of 148 days (~5 months). Glienke et al.,^6^ on the other hand, reported a longer median time to stricture diagnosis of 11.9 months, which may again be explained by their lengthy follow‐up. Although Elsaqa et al.^5^ did not report a specific time to diagnosis, they noted that half of strictures occurred within the first 6 weeks postoperatively and 20% after 6 months, despite a median follow‐up of 20 months. During follow‐up, we also found a recurrence rate of 23.5%, though there are no available data on recurrence of the previous studies. Only Taychert et al.^11^ noted that 50% of patients required multiple interventions, their analysis included bladder neck contractures and did not report stricture‐specific recurrence rates. Given that only four patients developed stricture recurrence after initial management, comparative or predictive analyses were not feasible at this sample size. Keeping this in mind, our observations are hypothesis generating only, and we do not intend to imply superiority of one treatment modality over another.

On multivariable analysis, higher BMI was independently associated with lower odds of postoperative urethral stricture. The relationship between lower BMI and increased stricture risk is not well characterized in the HoLEP literature. However, a large study evaluating urethral stricture after prostate surgery similarly identified lower BMI as an independent risk factor on multivariable analysis (HR = 0.990, *p* = 0.027).^17^ Notably, this cohort included both TURP (79%) and open simple prostatectomy (21%), with procedure type independently associated with stricture risk. Although these surgical approaches differ from HoLEP, the similar association between lower BMI and stricture risk suggests this factor may be relevant across different prostate surgery populations. Although the underlying mechanism remains unclear, these findings raise the possibility that patient physical characteristics may influence susceptibility to urethral strictures. Given the limited number of events in our cohort, this observation should be interpreted cautiously and viewed as hypothesis‐generating.

Increasing prostate size demonstrated a trend towards lower stricture risk but did not reach statistical significance. This relationship has been described in reports by Taychert et al.^11^ and Elkoushy et al.^18^; however, it is important to mention that Taychert et al.^11^ evaluated urethral complications as a composite outcome that included both bladder neck contractures and urethral strictures. Despite these differences in outcome definition, the consistent link between smaller prostate size and urethral complications suggests a potential underlying biological or anatomical susceptibility. Proposed explanations for increased risk in smaller glands include higher collagen content in prostates <50 g, which may predispose to scarring, as well as steeper bulbar urethral angulation, potentially increasing mechanical stress during surgery.^6^, ^19^ Overall, the relationship between prostate size and stricture formation remains unclear and warrants further investigation.

The predominance of bulbar strictures in our cohort (52.9%) aligns with previous reports, in which bulbar involvement has ranged from 33% to 90%.^5^, ^6^, ^11^, ^20^ This pattern supports the idea that mechanical stress is concentrated on the bulbar urethra, increasing susceptibility to trauma and subsequent scarring.^6^ As such, preventive strategies such as sheath size selection, preoperative urethral dilation and catheterization have been variably studied. Although some reports suggest larger sheaths may increase stricture risk, others have found no difference.^5^, ^11^, ^15^

Preoperative urethral dilation was associated with a lower stricture rate (0% vs. 6.45%) in a small series, and Glienke et al.^6^ reported a potential protective effect of preoperative catheterization, possibly through passive urethral dilation.^6^, ^21^ However, these findings have not been consistently replicated and were not observed in our cohort.^5^, ^11^, ^15^ In contrast, Elkoushy et al.^18^ found that preoperative catheterization was associated with urethral stricture formation and reported longer postoperative catheterization durations among patients who developed strictures; similar findings regarding postoperative catheterization were noted by Elsaqa et al.,^5^ although their analysis included BNC. These mixed findings suggest preoperative dilation may help in select cases, but further study is needed, particularly given that increased urethral manipulation could itself contribute to trauma. Our policy to do urethral calibration or Otis urethrotomy if a small size meatus or non‐distensible penile urethra was encountered during initial cystoscopy would have helped reduce the risk of meatal stenosis as noted by other authors.^22^

Limitations of this study include those inherent to its retrospective design and single‐surgeon experience, which affect generalizability. Unfortunately, we lack information on duration of preoperative catheterization in our database which might influence formation of postoperative urethral stricture. We also lacked complete data regarding use of intraoperative Otis urethrotomy in all patients. Follow‐up duration was variable, and some patients were lost to follow‐up, raising the possibility that late‐presenting urethral strictures were not captured.

AUA guideline (2023) defines urethral stricture as chronic fibrosis associated with narrowing of the urethral lumen that can be identified based on cystoscopic and/or radiographic findings.^23^ In the present study, evaluation for stricture was only performed in patients with LUTS or obstructed uroflow. Hence, it is likely that subclinical or asymptomatic urethral narrowing may not have been detected. In addition, the relatively low event rate may have limited power to detect independent associations with less common risk factors. Given the long study period and technique modification during the final years, trainee involvement and evolving surgical methods may have influenced urethral stricture outcomes. Finally, inclusion of patients with prior radiation therapy and prior transurethral prostate surgery—both recognized risk factors for stricture formation—may have introduced confounding, although their inclusion reflects real‐world practice.

Despite these limitations, the present study provides important contributions to the literature by specifically evaluating de novo postoperative urethral strictures, excluding preexisting and intraoperatively detected strictures as well as bladder neck contractures, thereby offering a more precise estimate of true postoperative risk. This is particularly relevant keeping inconsistencies in reporting of urethral strictures in mind (Table 2). In addition, we provide detailed characterization of stricture anatomy, timing and management outcomes, including recurrence after initial intervention, which has been incompletely described in prior HoLEP series.

## 5 CONCLUSION

The overall risk of urethral stricture following HoLEP was low (2%). Most strictures were <1 cm and located in the bulbar urethra. Higher BMI was independently associated with lower odds of urethral stricture, whereas smaller prostate volume demonstrated a non‐significant trend towards increased risk. Following initial intervention, 23.5% of men experienced recurrence requiring additional management. Our experience adds to the limited literature on this complication after HoLEP and would aid urologists in the shared decision‐making process.

## AUTHOR CONTRIBUTIONS

**Juanita Velasquez‐Ospina**: Protocol/project development; data collection or management; manuscript writing/editing. **Gurpremjit Singh**: Protocol/project development; data collection or management; manuscript writing/editing. **Ahmad Abdelaziz**: Protocol/project development; data collection or management; manuscript writing/editing. **Adam Williams**: Data collection or management; data analysis; manuscript editing. **Haşim Bakbak**: Protocol/project development; data collection or management; manuscript editing. **Jonathan Katz**: Protocol/project development; conceptual framework; manuscript writing/editing. **Robert Marcovich**: Protocol/project development; conceptual framework; manuscript writing/editing. **Hemendra N. Shah**: Protocol/project development; data collection or management; data analysis; manuscript writing/editing. All authors critically reviewed and approved the final version of the manuscript.

## CONFLICT OF INTEREST STATEMENT

The authors declared no potential conflicts of interest with respect to the research, authorship and/or publication of this article.

## Data Availability

All data supporting the findings of this study are available upon request.
